# Para sport and anti-doping: a study of Swedish Para athletes' experiences and perceptions

**DOI:** 10.3389/fspor.2024.1375359

**Published:** 2024-04-25

**Authors:** Anna Qvarfordt, Göran Svedsäter, Kristina Fagher, Anna Bjerkefors, Sven Blomqvist

**Affiliations:** ^1^ Department of Occupational Health, Psychology and Sport Sciences, Faculty of Health and Occupational Studies, University of Gävle, Gävle, Sweden; ^2^ Department of Health Sciences, Rehabilitation Medicine Research Group, Lund University, Lund, Sweden; ^3^ Department of Physiology, Nutrition and Biomechanics, The Swedish School of Sport and Health Sciences (GIH), Stockholm, Sweden

**Keywords:** anti-doping, policy, Para athletes, Para sport, survey

## Abstract

**Introduction:**

A well-functioning anti-doping system relies on being perceived by athletes as effective, fair, and practically feasible to implement. While research has highlighted the views of Olympic athletes on anti-doping over the past decade, the experiences and perceptions of Para athletes have not been extensively explored. The purpose of this study was to examine Swedish elite Para athletes' experiences and perceptions of the policy and practice of the anti-doping system.

**Methods:**

A quantitative cross-sectional approach was used, with a web survey elaborated from a survey with Olympic athletes adjusted for Para athletes with physical, visual, and intellectual impairments. The sample consisted of 66 active Para athletes competing at national or international level (response rate 71%). Data were analyzed using descriptive statistics and differences between subgroups were examined Fisher's exact test. Thematic analysis was employed to analyze open-ended questions.

**Results:**

Most of the respondents expressed a positive outlook on the anti-doping system, advocating for comprehensive efforts. A significant portion (35%) had not received anti-doping education, with those who did reporting increased confidence in avoiding unintentional doping. Despite their elite status, half of the respondents had not undergone doping control. Mistrust regarding the system's effectiveness and fairness was identified, with over half of the participants emphasizing the need for new technical solutions to enhance procedures specifically tailored for Para athletes.

**Discussion:**

The athletes in this study advocate for a Para sports-focused approach in the anti-doping system, emphasizing equal testing opportunities, procedural adjustments for independence and privacy, and increased access to education. The findings illuminate the unique conditions faced by athletes with impairments within the anti-doping system, offering valuable insights for policymaking in the development of anti-doping strategies tailored to Para athletes and their various impairments.

## 1 Introduction

Regulated anti-doping principles in elite sports are crucial for ensuring fair play for all athletes. To decrease and eventually eliminate the use of performance-enhancing drugs and methods, the anti-doping system must be perceived as effective, fair, and include measures that are feasible to pursue. Support from athletes, the main target for measures within the anti-doping system, is crucial for the system's functionality (1, 2) as the anti-doping system significantly impacts athletes' daily lives. For example, elite athletes must adapt their routines to comply with anti-doping regulations, often pushing the boundaries of their privacy. If athletes perceive these procedures as poorly adjusted, overly intrusive, or ineffective, it jeopardizes the system's functionality. While Olympic athletes' views on anti-doping policies have been studied in recent decades (2) Para athletes' perspectives remain largely unexplored.

Athletes participating in Para sports are categorized into three major types of impairments: athletes with physical, visual, and intellectual impairments. Para athletes face unique challenges due to their impairments, sometimes making anti-doping procedures difficult to handle which potentially can lead to feelings of exclusion in ethically sensitive situations. For instance, an athlete with physical or motor limitations, such as fine motor skill impairments, or reliance on a wheelchair, may require assistance during urine sampling. Visual impairment could impede an athlete's ability to follow and control testing procedures, and intellectual impairments can affect how regulations, education and procedures are perceived (3). Additionally, the greater need for medical drugs among Para athletes may conflict with medication regulations (4, 5). Test statistics also indicate that anti-doping rule violations are increasing in Parasport (6). Consequently, Para athletes often find themselves in situations that differ from their able-bodied peers, facing heightened exposure and dependence on others within the anti-doping milieu.

### 1.1 Anti-doping measures

The anti-doping rules in sports are global and based on efforts to prevent, detect, and sanction doping. Education, controls and granting of therapeutic use exemptions (TUE's) etc. are carried out by different actors at several levels within the sports context, all regulated by the World Anti-Doping Code (7), and its related International Standards. For underaged athletes and Para athletes, it is possible to modify measures such as sample collection procedures and equipment (7, 8). However, there are studies indicating that there are few adaptations implemented in the real-world sport setting. For example, Boardley et al. (9) studied anti-doping education for Para athletes and athlete-support personnel (ASP) and found that the design and delivery of educational programs are not adequately tailored to the requirements of this group. It is underscored that it is imperative to directly incorporate the specific needs of Para athletes and their ASP into relevant policies. Although some adjustments are made for Para athletes (7, 8), athletes’ physical, visual, or intellectual impairments can still hinder their independence, autonomy, and control in anti-doping measures (10).

### 1.2 Athletes' perspective on anti-doping

To ascertain the alignment of the anti-doping system with the specific requirements of athletes, it is imperative to solicit their direct input. A substantial proportion of research concerning athletes' viewpoints on the anti-doping system has been conducted in sports settings for able-bodied athletes [e.g., (11–17)].

Existing research on Olympic athletes shows that athletes support the principle of anti-doping, but that practical measures can be seen as ineffective, unfair or cause difficulties that could risk the athletes' willingness to pursue their obligations (1, 18, 19). For example, there has been criticism directed at the anti-doping system for its lack of efficacy and functionality (1). Furthermore, several high-profile doping incidents, such as the doping scandal in the Winter Olympics in Sotji, Russia in 2014 have also raised concerns over the effectiveness of the system to “catch the cheaters” (20). The elite athletes' views on the efficiency of anti-doping will most likely affect their confidence in the system.

Issues of integrity have also been discussed as a factor that could decrease the support for the anti-doping system. The urine sampling procedure puts the athlete in an exposed situation, and in studies among Olympic athletes the procedure has been found to cause feelings of stress and uncomfortableness about personal integrity (1, 21). Further, the whereabouts information system can entail integrity concerns as athletes may feel monitored and perceive negative feelings in their everyday life [e.g., (16, 22, 23)]. It has been discussed that the system for managing athletes’ tests and whereabouts information etc. possibly could infringe athletes’ privacy (15). Thus, integrity and privacy issues related to anti-doping procedures have been highlighted in research for athletes without impairment during the past decade.

The interest in also understanding Para athletes' perceptions of anti-doping policy and practice is beginning to grow. Weber et al. (24) showed in a qualitative study including elite Para athletes from Germany and UK that Para athletes perceive that doping occurs in Para sport, and that the anti-doping system does not work completely. The interviewed athletes were particularly distrustful of the TUE process. Furthermore, there were perceptions that anti-doping procedures, such as testing and education, are not carried out in the same way in different parts of the world (24). Blank et al. (6) conducted a survey examining the perspectives of Para athletes and Parasport coaches regarding anti-doping rule violations and responsibilities. The study revealed perceptions that anti-doping education was not provided to athletes as stipulated in regulatory documents. Additionally, there were perceptions of an unequal distribution and standard of such education on a global scale.

### 1.3 Research aim

Taken together, little is still known about Para athletes' view of anti-doping policy and practice (3), entailing a need for research targeting their experiences and perceptions of regulations and procedures, perceived fairness and effectiveness of the system as well as how anti-doping measures can progress. Therefore, the purpose of this study was to examine Swedish elite Para athletes' experiences and perceptions of the policy and practice of the anti-doping system. Specifically, the interest is directed at the athletes' view of anti-doping regarding (a) policy, (b) education and knowledge, (c) effectiveness and fairness, and (d) adaptations and new technology.

## 2 Methods

A quantitative cross-sectional approach was used to examine elite Para athletes' perceptions of anti-doping policy and practice in Sweden. The study follows the STROBE guidelines for epidemiological research.

### 2.1 Procedure

When designing the project, we assumed a methodological understanding that is responsive, which means that the project aimed to be democratic (25). A project group was established, consisting of two elite Para athletes (one with visual impairment and one with a severe neuromuscular impairment), one representative from Para sport Sweden and five researchers (each with their special knowledge in the field) from three universities in Sweden, to develop the project and the survey. To avoid a unilateral perspective emanating from the researchers' preconceptions and interests, special attention was paid to input from the athletes in the initial phase of the project. Based on their experiences in top-level Para sport, they emphasized difficulties that an athlete with an impairment can encounter, for instance, in an onsite doping control situation. They pinpointed issues such as exposure, accessibility, dependence and integrity as important in the development of anti-doping policy and practice, issues that were considered during the planning of this project. Participant involvement from all members in the project group has been fundamental for designing the study design, purpose, survey questions and variables as well as interpreting results.

### 2.2 Sample and data collection

The study sample consisted of active elite Para athletes with physical, visual and intellectual impairment competing at national or international level. In addition, younger Para athletes enrolled in Para sport Sweden's “Elite sports school” were invited to take part in the study. The Elite sports school consists of young promising Para athletes who are supported to take the next step in their sports career to reach the absolute top in their sport. The athletes for this study were recruited via the anti-doping officer at Para sport Sweden. In collaboration with coaches, a digital and accessible survey was distributed via email. Athletes were then given time to complete the survey at physical or online meetings for the national teams, to improve the chances for high response rates. Athletes from the following sports are represented in the survey: table tennis, judo, wheelchair rugby, para nordic skiing, para ice hockey, swimming, goalball, cycling, shooting, boccia and alpine skiing.

### 2.3 Questionnaire

The questionnaire was elaborated from a similar international survey assessing Olympic athletes' perceptions of anti-doping (1), and adjusted to be adapted for Para athletes with physical, visual and intellectual impairments. The adjustment was based on participant involvement (as described above), which resulted in several additional questions about how accessible anti-doping measures are for people with various impairments, whether the implementation of doping controls is adapted to the athlete's impairment, if technical solutions in anti-doping procedures are adapted to the impairment**,** whether the athlete finds it possible to provide a urine sample her-/himself without the help of the doping control officer/other person, etc. When the questions for the survey were drafted, a pilot survey was conducted including retired Para athletes (*n* = 3) to evaluate the survey content and the accessibility of the digital survey system. After that, minor adjustments were made. The final questionnaire consisted of the following areas: (i) background questions (sex, age, impairment, sport, years active in para sport); (ii) athletes’ experiences and perceptions of anti-doping policy; doping controls, whereabouts information, TUE and anti-doping education; (iii) questions about respect, trust, integrity and influence; (iv) and finally adaptations and accessibility of doping controls, policy and technical solutions. The questionnaire contained items of multiple choice-type and four-category response scale, e.g., ranging from “Strongly agree” to “Strongly disagree”. In addition, some of the questions had open-answer options.

### 2.4 Data analyses

A descriptive analysis was made to describe baseline characteristics and to assess experiences, opinions, perceptions and availability/accessibility of the anti-doping system. Differences between the views of different subgroups were examined with cross tables and Fisher's exact test (*p* < 0.05). Open-ended questions were categorized and analyzed, using thematic analysis (26). The use of the questionnaire by Efverström et al. (1) for Olympic athletes (adjusted) has allowed for comparisons between perceptions of Paralympic and Olympic athletes. The following subgroups were created: Those who have or have not undergone anti-doping education; those who considered themselves to have sufficient knowledge of the anti-doping (Strongly agree/Agree to some extent) and those who did not consider themselves to have that (Disagree to some extent/Strongly disagree). This was done to see if perceived knowledge and anti-doping education influenced the participants' answers. Subgroups were also created for those who had the experience of competing at national team/international level for five years or less and those who had that experience for six years or more. The cut-off for length of experience was set at five years as athletes in many sports retire between the ages of 25 and 30 on average and have an elite sports career that lasts around 10 years (27).

### 2.5 Ethical considerations

The project was approved by the Swedish Ethical Review Authority (Dnr 2021-05979-01) and follows the WMA Helsinki Declaration for research including human subjects. From an ethical standpoint, the risk of the study design and questionnaire content causing discomfort to the research participants has been assessed as low. Even though the benefit for athletes is not immediate, they may perceive it positively that their situation is being recognized. In the long run, improved anti-doping efforts will benefit the research participants as the study can provide a basis for policy development. Survey responses have been handled in such a way that no individual can be identified, and the risk of privacy infringement is minimized. Results are reported on group level.

## 3 Results

### 3.1 Athlete demographics

In the present study, 93 Swedish Para athletes received an invitation to participate. A total of 66 athletes (71.0%) accepted the invitation and completed the questionnaire. The demographic composition of the respondents reflected a predominance of male participants (72.3%), those possessing upper secondary school or university education credentials (81.8%), and individuals with physical impairments (76.9%). Furthermore, 58.5% of respondents were engaged in summer sports. Among the athletes surveyed, 52.3% were 26 years or older, with 53.0% having a competitive experience of six years or more at the elite level. Notably, only 16.7% had been granted a TUE, and 16.7% had filed whereabouts information. Detailed characteristics of the study population are presented in Table 1. Additionally, 50.8% of participants had not undergone doping control in connection with competition, while 70.8% had not been subjected to out-of-competition testing. The total number of athletes who underwent one or more doping tests was 32 (48.5%).

**Table 1:** Characteristics of the study group.

Sex	*n *= 65
Female	18 (27.7)
Male	47 (72.3)
Age	*n *= 65
20 years or younger	19 (29.2)
21–25 years	12 (18.5)
26–30 years	11 (16.9)
31–35 years	8 (12.3)
36–40 years	7 (10.8)
41–50 years	6 (9.2)
51 years and above	2 (3.1)
Impairment	*n *= 65
Physical impairment	50 (76.9)
Visual impairment	14 (21.6)
Intellectual impairment	1 (1.5)
Time of year	*n *= 65
Summer sport	38 (58.5)
Winter sport	22 (33.8)
Other sport	5 (7.7)
Year at elite level	*n *= 66
Never	5 (7.6)
1–5 years	26 (39.4)
6–10 years	20 (30.3)
10 years or more	15 (22.7)
Education	*n *= 66
High school	12 (18.2)
Upper secondary school	32 (48.5)
University	22 (33.3)
Granted a TUE	*n = 66*
No	55 (83.3)
Yes	11 (16.7)
Have filed whereabouts information	*n = 66*
No	55 (83.3)
Yes	11 (16.7)

*n*, number; (%), percent.

### 3.2 Anti-doping policy in general

A substantial majority of respondents, totaling 96.9% of the respondents agreed that doping controls are an important part of work against doping, and 65.5% agreed that the current sanctions for anti-doping rule violations are good or too mild. However, 32.8% could not or did not want to answer the latter question. Regarding anti-doping efforts in the future, 87.9% of the athletes think that it should be as comprehensive as today or even more. Furthermore, 81.8% of the athletes would like doping to remain prohibited (Table 2). Notable is that 13.7% think that doping should be allowed (either with or without medical supervision) in the future. Very few athletes (3%) are considering stopping with their sports because there are too much use of prohibited substances and methods. Similarly, almost no one (1.5%) is considering giving up their sport because anti-doping measures are too extensive. In the open commentary section, there were several athletes who expressed that they perceived that there are few anti-doping activities, and they called for extended efforts. One comment was: “*Feels like doping controls are not used as much in Para sports as with “not disabled” athletes. Para sports get a little “overlooked”.”* Another athlete would like to see more comprehensive testing: “*More out-of-competition tests for those who report whereabouts and tighter surveillance at national championships are needed.”* Thus, in general the athletes seem to be positive towards anti-doping activities and did not reject extended efforts against doping in Para sport.

**Table 2:** Anti-doping policy in general.

I think that doping controls are an important part of the work against doping in my sport	*n* = 64
Strongly agree	56 (87.5)
Agree to some extent	6 (9.4)
Disagree to some extent	0 (0)
Strongly disagree	0 (0)
Don’t know/Can’t answer	2 (3.1)
I think the current sanctions for anti-doping rule violations are	*n* = 64
Too mild	16 (25.0)
Good the way they are	26 (40.6)
Too hard	1 (1.6)
Don’t know/Can’t answer	21 (32.8)
I think that anti-doping work in the future should be	*n* = 66
More comprehensive than today	30 (45.5)
Just as comprehensive as today	28 (42.4)
Less extensive than today	0 (0)
Don’t know/Can’t answer	8 (12.1)
In the future, I think we should handle doping in the following way	*n* = 66
Doping should remain prohibited	54 (81.8)
Doping should be allowed under the supervision of a physician	5 (7.6)
Doping should be allowed	4 (6.1)
Don’t know/Can’t answer	3 (4.5)

*n*, number; (%), percent.

### 3.3 Education and knowledge

As many as 36.0% (*n* = 23) of the athletes indicated that they had not undergone anti-doping education, with no significant gender or impairment-related differences. Notably, the duration of an athlete's elite-level experience was found to be a determining factor in whether they had received anti-doping education, showing that athletes that had been at elite level for more than six years had received education to higher degree (*p* < 0.001) (Table 3). In the open commentary section regarding anti-doping education, one athlete expressed concern: “*It is problematic that education is not offered to athletes to a greater extent. For example, I, who have competed at an elite level for several years, have never been offered this education.”*

**Table 3:** Education and knowledge.

	Undergone anti-doping education		Fisher's exact test
	Yes	No	
Have sufficient knowledge of the anti-doping rules to avoid unintentional doping (*n* = 63^a^)			
Strongly agree/Agree to some extent	38 (92.7%)	15 (65.2%)	
Disagree to some extent/Strongly disagree	3 (7.3%)	7 (30.4%)	<0.016*
It is difficult to stay updated on the Prohibited List (*n* = 50^a^)			
Strongly agree/Agree to some extent	20 (52.6%)	9 (75.0%)	
Disagree to some extent/Strongly disagree	18 (47.4%)	3 (25.0%)	0.201
I have competed at national team/international level (*n* = 64^a^)			
0–5 years	12 (29.3%)	18 (78.3%)	
6 years and more	29 (70.7%)	5 (21.7%)	<0.001*

*n*, number; (%), percent.

^a^ Those who answered don't know/can’t answer are removed from the analysis.

* Significant (2-sided).

**Figure 1:**
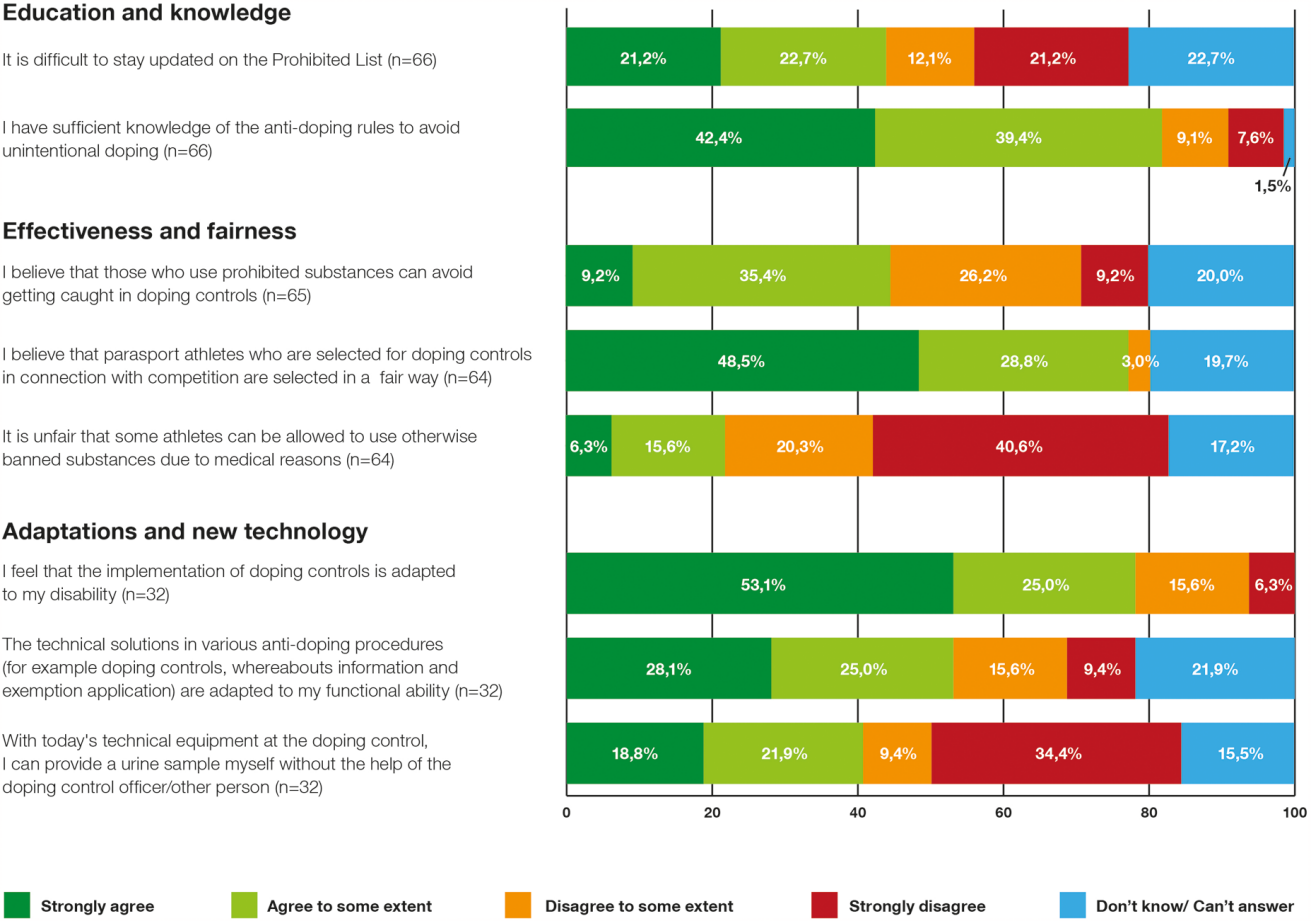
Summary of the area's education and knowledge, effectiveness and fairness and adaptations and new technology. Questions about Adaptations and new technology have been answered by those who had carried out a doping test (*n* = 32).

In Figure 1, the presented data indicates that a majority of athletes, accounting for 81.8%, believed they had adequate knowledge to prevent unintentional doping. Interestingly, those who had undergone anti-doping education exhibited significantly higher confidence in avoiding unintentional doping (92.7%) compared to those who hadn't received such education (65.2%), with a substantial statistical difference (*p* < 0.016) (Table 3). Regarding the perceived difficulty of staying updated on the Prohibited List, 43.9% found it challenging, while 22.7% could or would not provide an answer to this question, as illustrated in Figure 1. No significant differences between various groups were observed concerning the difficulties of staying updated on the Prohibited List (*p* < 0.201) (Table 3). In summary, a significant portion of the athletes in the study, even at elite level, have not received anti-doping education. Those who have received such education seem to have more confidence in avoiding unintentional doping. However, education does not appear to influence the perceived challenges in staying updated on the Prohibited List.

### 3.4 Effectiveness and fairness

Results showed that there is some skepticism concerning the effectiveness of the system, with 44.6% of the athletes expressing doubt whether doping controls can identify all those who use prohibited substances (Figure 1). Additionally, when asked about the prevalence of prohibited substance use among their competitors, half of the respondents estimated that 10% or fewer of their fellow contestants had engaged in such practices. Notably, a relatively large number of respondents (42%) either could not or chose not to provide an answer to this question. Most respondents (77.3%) appeared to view the selection procedure for doping control as fair (Figure 1). The perception of the fairness of athletes obtaining a TUE to use otherwise prohibited substances for medical reasons varied, with 60.9% not considering it unfair, while 21.9% found this practice to be unfair (Figure 1).

Some athletes raised concerns about the fairness and equity of the anti-doping system across different sports and nations. For instance, one athlete commented on the whereabouts information system, stating that it: “*seems to vary a lot between different sports regarding how many and at what “skill-level” athletes are required to file whereabouts information”.* Consequently, there are athletes who harbor doubts about the system's effectiveness and question its fairness in implementation to some extent.

### 3.5 Adaptations and new technology

Among the participants who had undergone a doping control, 78.1% expressed satisfaction with the adaptation of the doping control procedure to their impairments (Figure 1). Likewise, 53.1% found that the technical solutions encompassing procedures like the doping control, filing whereabouts, and applying for TUE were adapted to their functional abilities. Nevertheless, on the question “There is a need for new technological solutions that can facilitate the implementation of various procedures (such as doping control, whereabouts, and exemption application)”, 42.9% believed that new technical solutions are required to enhance anti-doping procedures for Para athletes. Regarding urine sampling specifically, 40.7% indicated that they couldn't complete the test without assistance using the current technical equipment, with no discernible differences between genders or athletes with various impairments (Figure 1). Several athletes commented on the challenges they faced during the urine sampling procedure. For example, one athlete with visual impairment noted: “*Athletes with visual impairments should be able to handle bottles themselves. Currently, we have to rely on the doping control officer or another person we bring with us.”* Similarly, another athlete remarked: “*It's uncomfortable to provide a urine sample in front of someone you don't know, which is compounded by the fact that I need assistance with the practical aspects of handling bottles and so on, due to my visual impairment. Even when someone I trust assists, it still makes me feel doubly restricted and uncomfortable.”* Additionally, an athlete with physical impairment suggested the need for a device to secure the cup during urine testing. In summary, a substantial number of participants who had undergone doping control found technical solutions in general accommodating to their functional abilities, but around half believed that improvements were needed. Especially, during the urine sampling procedure many athletes felt they required assistance due to current limitations.

## 4 Discussion

The purpose of this study was to increase the understanding of Swedish elite Para athletes' experiences and perceptions of the policy and practice of the anti-doping system. To summarize the results: almost all of those who responded were positive towards the doping control system, and many wanted anti-doping efforts to be as comprehensive as it is today or even more. Notably, one third of the participants had not received any anti-doping education, and those who had received education felt more confident in avoiding unintentional doping. A large proportion had not been selected for any doping control despite being an elite athlete. There was some mistrust about the effectiveness and fairness of the anti-doping system, and more than half of the participants expressed that new technical solutions are needed to better adapt doping procedures for Para athletes.

### 4.1 Perceptions of anti-doping policy

The study findings indicate a generally positive disposition among respondents towards anti-doping policy, which is in accordance with earlier studies among Olympic athletes [e.g., (1, 18)]. More than 90% of the Para athletes in this study acknowledged the significance of doping controls in the work against doping in sport, aligning closely with Olympic athletes, where a corresponding proportion of 91% was reported (1). Para athletes demonstrated a supportive stance on anti-doping policies, extending to their perspective on sanctions for anti-doping rule violations, with only 2% deeming the sanctions excessively harsh. Notably, approximately 33% either chose not to respond or were unable to answer this question. A comparison with Efverström et al.'s (1) study reveals similar trends among Olympic athletes, with 6% finding sanctions too severe, and 19% opting not to respond. Olympic athletes, possibly due to their familiarity with anti-doping regulations through education and experience, exhibited a greater ability to articulate their stance on sanctioning rule violations. Furthermore, 88% of the athletes in this study expressed support for maintaining or increasing the level of anti-doping efforts in their sport. Additionally, over 80% endorsed the continued prohibition of doping. This supportive attitude mirrors figures for Olympic athletes, where 80% favored sustaining or enhancing anti-doping efforts, and 77% advocated for continued prohibition (1).

Notably, only a very small percentage (1.5%) of athletes considered discontinuing their sports involvement due to the perceived extensive nature of anti-doping efforts. In the open commentary section, some athletes raised concerns about the perceived lack of anti-doping activities in Para sports, suggesting that they feel somewhat overlooked compared to able-bodied athletes. Calls for increased efforts were evident, with suggestions for more comprehensive testing, including more out-of-competition tests for athletes who report whereabouts and increased supervision at national championships. The underlying reasons for Para athletes' demand for more comprehensive anti-doping activities cannot be definitively determined. It may involve athletes viewing doping in Para sports as a real problem and requiring increased efforts to combat doping. The use of prohibited substances within Parasport indeed seems to be increasing. Zwierzchowski and Gaweł (28) emphasize that the growth of Parasport has led to heightened competitiveness, consequently raising the potential risk of unethical behavior. Furthermore, according to Blank et al. (6), who have analyzed test statistics from 2000 to 2019, the proportion of Anti-Doping Rule Violations (ADRVs) in sports overseen by the IPC has risen during those years. However, the results also suggest that Para athletes in this study perceive a lack of engagement and attention to doping issues within Para sports, interpreting this as their sport being considered less valuable. Thus, most athletes demonstrated a positive stance toward the anti-doping policy. Calls for increased efforts were evident, but the underlying reasons for Para athletes' demand for more comprehensive anti-doping activities remain uncertain.

### 4.2 Anti-doping education and athletes' knowledge

A majority of the participants thought they had sufficient knowledge to avoid unintentional doping. Despite this, almost half (44%) of the participants felt that it is difficult to stay up to date with the Prohibited List, which also has been seen in Olympic athletes (46%) (1). This is a concern as previous research has shown that 49% of Swedish elite Para athletes use some prescribed medication, and 22% regularly use supplements (29). Subsequently, it could be recommended to better provide education to Para athletes on when and how to use the Prohibited list.

Results from this study also showed that over a third of the respondents had not participated in anti-doping education, and these participants also felt much more uncertain about whether they had sufficient knowledge to avoid unintentional doping. A study by Blank et al. (6) revealed that over 33% of Paralympic athletes' first contact with the anti-doping system was an actual doping control and not anti-doping education. This is aligning with our results, which is a concern. According to the World Anti-Doping Agency's (WADA's) guidelines, the first contact should occur through anti-doping education (30). The responsibility for offering education in the anti-doping system to athletes is a collective responsibility between national anti-doping organizations (NADO), WADA, and the national sport federations. In the previously mentioned study, it emerged that athletes primarily received their education through NADO, followed by the national sport federations, with WADA ranking third. In the future, it is important to distribute the responsibility for education among these three different organizations to ensure that athletes are not overlooked in terms of anti-doping education. It is especially important to organize education that is adapted to Para athletes, i.e., to athletes with visual impairment and intellectual impairment, which also has been suggested in previous study (9). Additionally, providing the necessary resources to fulfill the three organizations' mission is crucial.

### 4.3 An effective and a fair system?

Many of the respondents in this study had never conducted an in-competition (50%) or out-of-competition (70%) doping test, and over 40% responded that doping controls do not catch everyone who uses prohibited substances. This is a concern as many of the participants in this study are elite athletes, and the results indicate that doping controls in Para sport occur seldom which should be seen as a concern for both NADO and WADA. Notably, over 95% agree that doping controls are an important part of the anti-doping system. The results show that even though many athletes have little or no experience with doping controls, they believe that doping controls are needed, that they are not effective enough and that anti-doping efforts do not adequately catch those who use prohibited substances. Perceptions of shortcomings in the system's effectiveness could stem from elite athletes' infrequent or non-existent testing experiences, leading them to doubt whether the system effectively catches cheaters. Other studies of Olympic athletes confirm that the system is perceived as ineffective [e.g., (1, 18, 31)]. One reason why athletes in general do not fully trust the anti-doping system may depend on the fact that there have been several doping cases that have been organized by different organizations, such as the doping scandal in the Winter Olympics in Sochi, Russia in 2014. Another reason why Para athletes are skeptical of the effectiveness of the anti-doping system may be that there are few doping controls carried out in Para sport, and this lack of experience with doping controls may influence their perception of the system. Blank et al. (6) show that few doping tests are carried out in Para sport, and that knowledge of anti-doping is very limited which the result in this study also indicates.

Most of the respondents in this study believed that the selection of athletes for doping control in connection with competitions is based on a fair manner. Athlete selection is an important part of the doping control process, and athletes’ perception of this process is an important prerequisite for an effective and targeted anti-doping system. Indeed, it is interesting that the athletes believe that the doping controls do not “catch the cheaters” but at the same time feel that the selection of athletes for doping control is a fair procedure. One possible explanation for a more positive perception of the selection process may be that in connection with competitions, a professional anti-doping organization is responsible for the administration, selection of athletes, notification, and doping control.

When asked whether athletes can be exempted from using banned substances for medical reasons, around 60% responded that this is not unfair. This result indicates that athletes feel that the TUE process is handled fairly, which is important for trust in the anti-doping system, especially in Para sport since many athletes use some medication related to their impairment (4, 5, 29). The fact that over one fifth of the respondents believed that the TUE regulation is unfair should also be noted. It is difficult to determine whether these perceptions are based on negative experiences with the TUE system or if they may be attributed to a lack of insight into the procedures for exemptions. If the latter, this could possibly be addressed with athletes’ full access to education.

WADA's policy documents [the Code, International Standard for Testing and Investigations (ISTI), etc.] often refer to effectiveness and fairness. One of the foundational principles in sports is the “Spirit of Sport,” wherein fair play is frequently emphasized as a crucial element. As regards doping, fair play can be seen as encompassing both the efficiency of the anti-doping system, ensuring that athletes do not have to compete against individuals who have used prohibited performance-enhancing substances, and the equitable and consistent implementation of anti-doping efforts across all sports globally (1). In the context of this study, Para athletes appear to assert that the system is not entirely effective in preventing doping and is not fully tailored to their specific circumstances. Although the study has only highlighted a few aspects of effectiveness and fairness, this information is nonetheless significant, contributing to a more comprehensive understanding of athletes' perspectives on anti-doping policies which could further be beneficial to increase the credibility of the anti-doping system.

### 4.4 Abilities, impairments, and new solutions

In this study almost half of the respondents had undergone a doping control (49%) A relatively large proportion of those athletes stated that the technical solutions in various anti-doping procedures generally are adapted to their functional abilities (53%). The procedure that seems to be of most concern is the doping control procedure, especially the urine sampling. In addition almost half of the athletes who had undergone doping control stated that new technical solutions are needed, and the written comments from the athletes shed light on what would improve the doping control situation for athletes with impairments. Suggestions included the ability to independently handle urine sample bottles (e.g., with braille) for individuals with visual impairments. Additionally, there were proposals for the development of devices to assist athletes with limited function in their arms and hands while providing a urine sample. Taken together, the results from this study show that there is a need for more independence and privacy during the urine sampling procedure. As Zwierzchowski (10) emphasizes, there are distinct differences between Paralympic and Olympic athletes, and there is a need to better adjust anti-doping regulations to the unique characteristics of Para athletes. In the context of anti-doping, having an impairment can present challenges, partly because the procedures were not originally designed with a primary focus on para-athletes. According to regulatory documents from WADA and the International Paralympic Committee (IPC) (7, 8) it is possible and recommended to adjust anti-doping regulations for Para athletes but judging by the findings in this study there is still work to be done to better suit the conditions for this important group of elite athletes.

During the past decades new technology and innovation have continually played an important role in the advancement of the anti-doping system, often in terms of new methods to detect doping (32, 33). Based on the results from this study we also recommend that new technology and innovation be used to improve and assure legal and autonomous anti-doping procedures for athletes with various impairments. Persons with an impairment are often used to using different types of assistive technology, and the development of new systems adapted to Para athletes could contribute to a more fair and inclusive anti-doping system.

### 4.5 Limitations and strengths

A limitation of this study may be that the questionnaire used originally was developed for Olympic athletes (1). Simultaneously, this procedure allowed for comparisons between Olympic and Para athletes, and it is a strength that the questionnaire was adapted to Para sport in collaboration with Para athletes, representatives of Parasport Sweden and researchers to increase the content validity of the questionnaire for the study group. By using a responsive and democratic process, all members in the project group had an influence on the project. Involvement from all parties have entailed fundamental inputs and discussions on the purpose of the project, information desirable to collect, questions to ask and the analysis of the results. The process was important to avoid the researcher's preconceptions and interests alone. After that we tested the questionnaire on former elite Para athletes to strengthen the validity and reliability of the questionnaire and investigate how accessible the questionnaire was to different impairments. Then some minor changes were made to clarify certain questions and increase the accessibility of the questionnaire. The aim of this process was to increase the study's internal validity.

The response rate can be considered high (34), which strengthens the external validity of the study's results. What is also positive for external validity is that many different sports, both individual and team, summer and winter sports are represented in the study group. Something that reduces the study's external validity is that many athletes answered that they didn't know or couldn't answer several questions. One interpretation of this phenomenon is that the respondents didn't feel confident they wouldn't be identifiable. In such a scenario, individuals may have hesitated to provide answers that diverged from prevailing anti-doping norms, leading to a reluctance to respond to certain questions. It is worth noting, however, that the data was anonymized to ensure participant confidentiality, a fact explicitly communicated to all participants prior to their involvement in the study. Alternatively, it is conceivable that the relatively high proportion of respondents who gave the answer “don’t know/can’t answer” could be attributed to factors such as limited exposure to anti-doping education [cf (9).]. Additionally, the youthfulness of the participant cohort and their relatively limited experience with doping controls, filing whereabouts information, and applying for TUEs may also contribute to this trend.

A limitation in this study is that only one athlete with intellectual impairment participated in this study, meaning that the results cannot be generalized to this group. Furthermore, the survey, in general, has a relatively small number of participants, which means that it is not possible to divide the data into different subgroups to investigate potential differences in perceptions of anti-doping among groups such as women/men, impairments, etc. Another limitation is that only Swedish Para athletes from a high resourced setting are included in the survey, which makes it difficult to generalize the results to Para athletes globally. Thus, it is recommended to include athletes from various resourced settings in future studies.

### 4.6 Conclusion

Para sport is experiencing a significant growth and impact both within the sports community and society (24). For example, there is an increasing number of athletes, greater media attention, and a growing economic presence. Furthermore, elite Para athletes' performances have increased tremendously in the past decade. These are factors that may contribute to the use of prohibited substances to improve performance and success, and as mentioned, statistics do indicate an increase of anti-doping rule violations (6). Hence, there are indications that doping is not less occurring in Para sport than in other sports. Importantly, results from this study show that many Para athletes have not ever been selected for a doping control despite being an elite athlete competing at international level. Considering these observations, it is reasonable to argue that the “doping issue” in Para sport should be addressed equally seriously and thoroughly as it is in sports for athletes without impairments.

The study sheds light on the conditions for athletes with an impairment in the anti-doping system, and the results can contribute to policymaking of the development of anti-doping strategies adapted to Para athletes and their various impairments. To further enrich our understanding, it would be beneficial to expand the investigation to include an international perspective, and especially target Para athletes with intellectual impairments, as emphasized by Hurst and Burns (3). To delve deeper into the nuances of this subject, a larger and more diverse sample would be necessary, allowing for a more detailed exploration of potential variations in opinions among various subgroups. Moreover, the results indicating that new technology and innovation can enhance autonomous procedures for Para athletes pave the way for applied research in close collaboration with the athletes themselves. Finally, adopting a qualitative research approach would provide a more profound comprehension of the perceptions and challenges that Para athletes encounter within the anti-doping system.

## Data Availability

The raw data supporting the conclusions of this article will be made available by the authors, without undue reservation.
